# UK ethnicity data collection for healthcare statistics: the South Asian perspective

**DOI:** 10.1186/1471-2458-12-243

**Published:** 2012-03-27

**Authors:** Gulnaz Iqbal, Mark RD Johnson, Ala Szczepura, Sue Wilson, Anil Gumber, Janet A Dunn

**Affiliations:** 1 Warwick Clinical Trials Unit, Division of Health Sciences, Warwick Medical School, The University of Warwick, Coventry, UK; 2 Centre for Evidence in Ethnicity Health and Diversity, University of Warwick, Coventry, UK; 3 Mary Seacole Research Centre, De Montfort University, Leicester, UK; 4 Primary Care Clinical Sciences, University of Birmingham, Birmingham, UK

**Keywords:** Ethnicity, Data collection, Perspectives, South Asians, Focus groups

## Abstract

**Background:**

Ethnicity data collection has been proven to be important in health care but despite government initiatives remains incomplete and mostly un-validated in the UK. Accurate self-reported ethnicity data would enable experts to assess inequalities in health and access to services and help to ensure resources are targeted appropriately. The aim of this paper is to explore the reasons for the observed gap in ethnicity data by examining the perceptions and experiences of healthy South Asian volunteers. South Asians are the largest ethnic minority group accounting for 50% of all ethnic minorities in the UK 2001 census.

**Methods:**

Five focus groups, conducted by trained facilitators in the native language of each group, recruited 36 South Asian volunteers from local community centres and places of worship. The topic guide focused on five key areas:1) general opinions on the collection of ethnicity, 2) experiences of providing ethnicity information, 3) categories used in practice, 4) opinions of other indicators of ethnicity e.g. language, religion and culture and 5) views on how should this information be collected. The translated transcripts were analysed using a qualitative thematic approach.

**Results:**

The findings of this Cancer Research UK commissioned study revealed that participants felt that accurate recording of ethnicity data was important in healthcare with several stating the increased prevalence of certain diseases in minority ethnic groups as an appropriate justification to improve this data. The overwhelming majority raised no objections to providing this data when the purpose of data collection is fully explained.

**Conclusions:**

This study confirmed that the collection of patients' ethnicity data is deemed important by potential patients but there remains uncertainty and unease as to how the data may be used. A common theme running through the focus groups was the willingness to provide these data, strongly accompanied by a desire to have more information with regard to its use.

## Background

Over recent years there has been a drive for improved ethnicity data collection from the National Cancer Inequalities Initiative and National Cancer Intelligence Network with the main Hospital Episode Statistics (HES) data being scrutinised for completeness and validity [1,2]. Inequalities in health and access to healthcare according to ethnic group have been reported; this is of particular concern in cancer where Black, Minority and Ethnic (BME) patients have been shown to have differing rates of certain cancers compared to the general population [3-12]. A recent study showed women of African-Caribbean origin to have higher rates of breast cancer compared to the UK white population [5]. Disparities in the incidence of prostate cancer have been apparent for many years resulting in the recommended age for Black-American men to commence screening to be lowered from 50 to 40 years in the USA [13]. However, these inequalities are not restricted to cancer, disparities by ethnic group have also been observed in diabetes, where South Asians are six times more likely to develop diabetes and coronary heart disease than the general population [4]. South Asians are the largest ethnic minority group accounting for 50% of all ethnic minorities in the UK [14]. Despite looking similar in outward appearance they differ greatly in terms of their culture, religion, language and diet.

Ethnicity data is generally known to be incomplete and of poor quality in the NHS with many still unaware of the importance of the data and its uses. Without reliable ethnicity data it is not possible to investigate differences between groups further or to develop strategies to tackle inequalities [2,15]. In 1995 it became Government policy in England and Wales to record ethnicity in Hospital Episode Statistics (HES) and in secondary care, and although there have been some great improvements such as the decline of not known/not stated codes in Finished Consultant Episodes from 23.9% in 2004-05 to 8.6% in 2009-10, HES data remains incomplete [16]. The Quality and Outcomes Framework (QoF) began awarding points (linked to financial incentives) to GP practices collecting ethnicity data on all newly registered patients in 2003. Furthermore, the collection of ethnicity data has been actively encouraged in healthcare for many years. In 2005, the Department of Health produced 'A practical guide to ethnic monitoring in the NHS and social care' which explained the relevance of data items and provided examples of good practice [17]. The drive towards the collection of complete and reliable ethnicity data stems primarily from the passing of the Race Relations (Amendment) Act (2000) which places responsibility on authorities to not only minimise inequalities but to actively promote equality. October 2010 saw the amalgamation of anti-discrimination laws to form a super Equality Act prohibiting discrimination on the grounds of nine characteristics inclusive of race and religion or belief [18].

A limited amount of research has been conducted in this area internationally, primarily in the USA. In one study of patients' attitudes towards healthcare professionals collecting data on ethnicity and race, Baker reported over half the study population to be either somewhat or very concerned (51.2%) that the data would be used to discriminate against them [19]. This proportion was significantly higher in participants of Black/African American origin compared to those of White origin (74.3% vs. 40.9% respectively).

In 1996, soon after the initiation of the mandatory ethnicity data collection in secondary care, Pringle and Rothera showed ethnicity data collection to be feasible as well as acceptable to patients and staff in the primary care setting [20]. More recently, the Information Services Division, Scotland successfully demonstrated the feasibility of collecting extra personal data (including ethnicity) for all new registrations [21]. However, there has been limited new information on how healthcare professionals and members of the public in the UK perceive ethnicity data collection despite moves to improve the completeness and reliability of ethnicity data.

The aim of this research is to explore barriers to ethnicity data collection by evaluating the perceptions and experiences of BME participants and their willingness to provide this information, investigated through a series of focus groups conducted with healthy volunteers. South Asians are the largest minority group making up 4% of the total UK population and 50% of the UK's total non-white population in 2001 [14]. Despite a similar outward appearance people originating from South Asia (most commonly India, Pakistan, and Bangladesh) are heterogeneous in terms of culture, language, religious beliefs, diet, migration history, educational attainment and social class. In order to tackle the issues of incomplete ethnicity data in health care we need to consult with these groups to not only gather their views and experiences of data provision but also on the adequacy of the fields and categories generally utilised.

This work follows on from a systematic literature review of ethnicity data collection methodology in primary and secondary care [22]. This was conducted as part of a Cancer Research UK commissioned feasibility study to improve ethnic data collection for statistics of cancer incidence, management, mortality and survival in the UK. 'Barriers to collection' was one of seven main themes identified by the systematic review and revealed healthcare professional and patient perceptions to be major obstacles to the collection of ethnicity data. Fear of causing offence to patients or encountering resistance along with confusion about ethnicity categories and a lack of understanding of the need for ethnicity data were reported as deterrents by healthcare professionals in two reports from the USA [23,24]. This paper aims to identify barriers to data collection and reports the perceptions and experiences of South Asian participants originating from Pakistan, India and Bangladesh of providing these data.

## Methods

Focus groups conducted in the native language of each group was deemed the most appropriate method for this feasibility project with limited time and resources. In addition, it was felt that focus groups would allow discussion and debate between participants in what some may feel is a sensitive area.

The focus groups were conducted in collaboration with the Mary Seacole research centre at De Montfort University and the Ethnic Health Forum in Manchester. A topic guide was developed by the project team and ethical approval was obtained through South Birmingham LREC (ref: 07/Q2707/33) awarded March 2007). Focus groups were conducted by trained facilitators who recruited volunteers from local community centres and places of worship (5-10 participants per group), where the meetings also took place. Conducting the discussions in surroundings familiar to the participants was deemed essential to create a relaxed and informal atmosphere where participants would not feel intimidated, thereby encouraging open discussion. Gender segregation was observed as per cultural custom for the Bengali and Urdu speaking participants.

Incentives were offered to encourage participation. Facilitators selected the incentive they judged would be most effective in attracting their local population. The older Bengali group were provided with refreshments including lunch after the discussion whilst the Urdu, Mirpuri and Punjabi groups received payment in the form of high street vouchers. Informed consent was taken by the facilitator where English was not the volunteers' preferred language. The facilitators used the topic guide which was specifically developed to focus on five key areas:

1. General opinions on the collection of ethnicity

2. Experiences of providing ethnicity information

3. Categories used in practice

4. Language, Religion and Culture

5. How should this information be collected?

See additional file 1 for full topic guide.

All sessions were recorded, transcribed and translated by the facilitators. Additional notes on the conduct of the groups were taken by the moderator. Each focus group discussion was subject to a quality check by an independent reviewer who listened to the recordings and validated against the translated English summary. The recordings were listened to in full and the translated transcripts provided by the facilitators were reviewed. The translated transcripts were analysed using a qualitative thematic approach which involved examining the data, comparing the accounts with one and another and identifying common themes. Themes were developed and discussed by the project working group.

## Results

Five focus groups were conducted by trained facilitators, each speaking in the preferred language of their group and also in English if required. The number of participants in each group ranged from five to ten, with 36 participants in total. Across groups, there was an even number of males and females. The Bengali males were the oldest group, whilst the Urdu females were the youngest (median age 63 vs. 28.5 years respectively). Data on age were not available for the Mirpuri group. A great deal of discussion in the young Urdu females group took place in English since all members had a high standard of English. For the remaining groups, the native language of the group was used in order to include all participants in the discussion. The characteristics of the total 36 volunteer convenience sample are shown in Table 1.

**Table 1:** Characteristics of participants

Group	Country of origin	Language	Gender		Median age (range)	Total
			**M**	**F**		
1	Azad Kashmir	Mirpuri	0	5	-	5
2	Bangladesh	Sylheti/Bengali	8	0	63 (45-70)	8
3	Pakistan	Urdu/English	0	10	28.5 (18-35)	10
4	Pakistan	Urdu	8	0	30 (24-44)	8
5	India	Punjabi	2	3	31 (26-51)	5

**Total**			**18**	**18**	**31.5 (18-70)**	**36**

## 1. General opinions on the collection of ethnicity

In general, participants thought that accurate recording of ethnicity data was important. The majority were proud of their origins and were familiar with the differences between their's and other cultures, and understood the potential utility of such data in a healthcare setting. Several were also aware of the increased prevalence of certain diseases in minority ethnic groups and stated this as a reason supporting ethnicity data collection in a healthcare setting:

• *"Sometimes it is helpful to provide ethnicity as it helps care providers understand our background and determine common illnesses due to dietary habits or genetic findings... However, we should be told why it is being collected when asked for it" *[Punjabi female]

• *"Sometimes certain illnesses are directly linked to our ethnicity... For example stroke or diabetes..." *[Urdu female]

• *"... say you have diabetes, they want to know how many Bangladeshis suffer from diabetes, why they suffer from diabetes; how many Pakistanis, how many Somalis. Later they total up these figures to obtain another figure - the percentage for South East Asians altogether..." *[Bengali male]

A number of participants mentioned the importance of monitoring access and uptake of services whilst others mentioned the need for collection of ethnicity for future planning. Younger participants in particular felt that it was acceptable to provide ethnicity data for health purposes but not for other reasons such as job applications:

• *"It could be alright with diseases but when you have to give this information while applying a job it would be felt like discrimination..." *[Urdu female]

• *"It differs according to situation like if we are going for health service then it is acceptable as we are also getting some services in return but I don't see any point of providing information for employment purposes" *[Urdu male]

A small proportion (4 out of 36) did not understand the need for ethnicity data collection as they did not think it was relevant to treatment, or felt that they may be discriminated against if ethnicity was given:

• *"Because ethnicity should never be a deterrent or an incitement when it comes to service or health provision so there's no reason for why it should be collected" *[Mirpuri female]

• *"Because we are all human and the same and so our ethnic origin should not interfere with the care we receive..." *[Punjabi female]

• *"It is important for government point of view but there is no importance from our point of view" *[Urdu male]

When asked whether they had any objections or worries about providing ethnicity data the majority had no objections. Several had concerns related to feelings of discomfort if the purpose of data collection was not fully explained, and expressed fears of being stereotyped. There was dissatisfaction that the appropriate ethnicity category sometimes did not appear on the form, and there was also a feeling the data would not be utilised. One participant did not think discrimination was a problem given the multi-cultural make-up of the NHS workforce:

• *"I feel uneasy sometimes and you start wondering why they ask me questions about my ethnicity" *[Urdu male]

• *"Sometimes patients may not be treated as individuals, we may judge by ethnicity and assume they have this problem as its high in their group" *[Mirpuri female]

• *"My only problem is when the category is not available on a form, e.g. British Asian, I very rarely see this category. However, I have no problems as the information is confidential and most of the time nothing is done with information apart from stored on their files for years to come" *[Punjabi female]

• *"The NHS is so large with multi-cultural staff that I am not concerned I will be discriminated if my ethnicity is collected. However, I feel they should tell us when the information is collected and what it will be used for" *[Punjabi female]

### 2. Experiences of providing ethnicity information

In general, when asked about their experience of providing information about their ethnicity, the majority of people found it acceptable. Others expressed dissatisfaction about being asked to provide their ethnicity on repeat visits. The majority wanted some explanation as to why the data was being collected and what use it would be:

• *"No one tells us why are they asking such questions and I feel they should tell me why do they need this information" *[Urdu male]

The main reason given for negative experiences was inappropriate codes for recording ethnicity and the fact that on several forms they would be coded as 'other', which led to feelings of frustration and insignificance:

• *"When I have to state 'Other' as my ethnicity is not on the form and I feel even now my origin is not widely recognised" *[Punjabi male]

• *"Most forms did not differentiate Asians, as Asian can be different groups, and not just Pakistani, not just Chinese, also people are living in Kashmir part of Pakistan do not like calling themselves Asian Pakistani, but want to be grouped as Asian Kashmiri, and recently that has been acknowledged" *[Mirpuri female]

None of the participants had an objection to providing ethnicity information in a healthcare setting. However, there was some confusion about ethnicity data collection procedures in healthcare and the need for standardisation:

• *"Sometimes they ask these questions about ethnicity and sometimes they do not so we are not sure what is the standard routine" *[Urdu male]

• *"My child was born in the same hospital yet they ask ethnic data about him whenever I took him to hospital" *[Urdu male]

### 3. Categories used in practice

When discussion was focused on categories used in practice to describe individuals, many participants wanted country of birth, language and religion to be collected, in order to be able to distinguish between 'South Asians'. One participant thought that additional information on diet was useful; another participant also thought it would be helpful if individuals were asked whether or not they wanted to be donors:

• *"The current ones are fine but language would be good as there are cultural differences depending on what language you speak" *[Punjabi male]

• *"My background is I am from Bangladesh, so British Bangladeshi, this is fine. My son was born and brought up here, so he will say British - that's it" *[Bengali male]

• *"British Bangladeshi gives them accurate information for research [this was supported by two more participants]. For political reasons I say 'British Muslim', When it comes for ethnicity for medical research I would say British Bangladeshi" *[Bengali male, most of the others in the group agreed with him]

• *"The ethnicity should not be confused with the colour of the skin" *[Urdu female]

### 4. Language, religion and culture

Overall, all participants were happy to disclose their religion and language as long as they did not perceive that they were being stereotyped. The discussion on culture centred on religion being a better indicator of culture than 'ethnic group'.

• *"I have been asked, I have provided only because I'm not ashamed of my religion and whether I mind would depend on why I'm being asked" *[Mirpuri female]

• *"I would not hesitate to describe my language as Bengali, no reason to feel "sonkuchito" ["sense of shame"- others agreed with him]" *[Bengali male]

• *"Religion should be a part of ethnicity because that is the base of one's lifestyle and dietary requirements. We do not know if the medicines we are taking are in accordance with the dietary requirements of our religion e.g., most of the cough medicines may have alcohol in them" *[Urdu female]

• *"Language is important because sometimes an interpreter may be required..." *[Urdu female]

Some Muslims did feel that they were stereotyped, especially with the heightened awareness of terrorism:

• *"Fear of stereotyping is there. Any brown complexion person may be called a Paki or a girl with head scarf may be labelled a terrorist. This is the main fear of disclosing one's origin" *[Urdu female]

• *"There is always that risk in everyday life, but I guess people are far too busy with other duties to take notice" *[Mirpuri female]

• *"Yes, I feel that I am regarded as a vulnerable women because I am a non-English speaking person" *[Punjabi female]

• *"I am not Pakistani, I am a Bangladeshi. Because of my colour and appearance someone is calling me "Paki". This is stereotyping" *[Bengali male]

• *"The suspicion is that all Muslims are terrorist. This is a stereotyped view. This kinds of stereotype views should not be allowed" *[Bengali male]

Stereotyping by healthcare staff was also an issue for some participants:

• *"Walk-in centres provide independent advice but I feel my GP knows my family history so makes assumptions about me" *[Punjabi male, participant 3]

### 5. How should information be collected?

The Bengali focus group summarised how information should be collected:

• *"They should explain why they collect the data; the reason behind it; what benefit there will be for people. Also, where the data will be used and how secure this data will be. It should be kept secret [confidential]" *[Bengali focus group; all participants]

Most participants agreed that GPs should collect ethnicity data once and that this should be available to hospitals. There was a general consensus that not enough information is provided as to the use and importance of this data. When asked about routine data collection there was a strong feeling that the data should not be collected every time as information relating to ethnicity is not likely to change very often if at all e.g. religion:

• *"No way. There is no need for routine collection. If it really has to be it only needs to be collected once at each institution" *[Mirpuri female, participant 1]

• *"The information should be collected at the GP surgery as patients are already distressed in hospital" *[Punjabi female, participant 1]

In summary, the majority of focus group participants had no objections to providing the data but a brief explanation of the reasons for the data collection was considered highly desirable.

## Discussion

The principal findings of this Cancer Research UK commissioned feasibility study to improve ethnicity data collection for cancer statistics overwhelmingly indicates that there was no objection to providing ethnicity data for healthcare purposes in this South Asian population of focus group participants. A number of participants confidently demonstrated an understanding of differences in disease patterns by ethnic group and highlighted this as the main reason why collecting accurate ethnicity data in healthcare is of the utmost importance. There was also a consensus that ethnic group in isolation is not sufficient to capture the multi-faceted concept that is ethnicity. Many wanted additional data items such as country of birth, language and religion to be collected in order to distinguish between South Asian populations. The majority were proud of their origins and were familiar with the vast cultural differences between themselves and other South Asian communities. A small number of participants had reservations about providing the data and expressed feelings of discomfort when the purpose of the data collection and its intended use was not fully explained. Several participants expressed feelings of frustration when their ethnic group did not appear on the form and they had to tick 'other' whilst others objected to repeating the same information at every hospital visit. Most agreed GPs should collect ethnicity once and this data should be linked to hospitals. A few participants worried about disclosing their ethnicity fearing they would be labelled as terrorists, however, the majority of participants did not feel stereotyping was a problem.

This research was conducted as part of a Cancer Research UK commissioned feasibility study to improve ethnicity data collection for cancer statistics and was limited in terms of time and funding. Nevertheless, we were able to concentrate efforts on the largest minority group, South Asians made up 50% of the UK's total non-white population and 4% of the total UK population in 2001 [14]. In accordance with the cultural custom of gender segregation the Bengali, Mirpuri and Urdu speaking groups were conducted for males and females separately, further to this, same gender facilitators were also sought for each group. Unfortunately, we were not able to find a Bengali speaking female facilitator or a male Mirpuri speaking facilitator in the timeframe of the project.

Interpretation of the findings reported here should take into account the purposeful sample and the voluntary nature of the participants, therefore this sample may be biased in favour of providing ethnicity data and results may not be generalisable to other British South Asians. To our knowledge there is little information on the perceptions of ethnicity data collection for healthcare in the UK. In contrast much has been done in the area of ethnicity data collection in the USA where the proportion of ethnic minorities is greater [25]. Despite its limitations this research has provided important messages which can be used to inform future policy and advocate the need for accurate collection of these data. The findings could also be incorporated into staff training programmes to dispel barriers to collection and address common qualms such as the fear of offending patients.

Much research into improving ethnicity data collection has been conducted in the USA where the composition of the population and healthcare systems are very different to that of the UK. The findings presented here are rich and provide a detailed picture of the views of British South Asians, building upon Pringle and Rothera's investigation into the area 15 years ago and Baker's more recent exploration of patient's attitudes to ethnicity data collection in the USA [19,20,26]. Other published work in this area includes studies reporting the feasibility of automated data linkage whereby data collected in primary care is linked through to secondary care eradicating the need for repeated collection [20,21,27].

The majority of participants considered that a brief explanation as to why the data was needed and how it would be used would increase willingness to provide ethnicity, neglecting to offering an explanation or simply telling patients it was 'routine' or 'procedure' was not deemed satisfactory. There was a strong feeling amongst some participants that data collected for 'statistical purposes' is not utilised. These findings concur with those of Pringle and Rothera and Hasnain-Wynia et al who concluded patients must be told the reason for collection and the resulting data would be used to improve the quality of services for patients [20,28]. Ultimately, evidence of data use in healthcare and government reports may be the catalyst needed to improved ethnicity data collection.

Focus group participants also stated that staff should appear comfortable when asking questions about ethnic origin. Discomfort exhibited by members of staff could make patients suspicious of the motives behind the questions and exacerbate non-compliance. Baker et al reported changes in patient comfort levels providing race and ethnicity data after hearing four different rationales, 1) quality monitoring, 2) government recommendation, 3) needs assessment and 4) personal gain. Comfort levels were shown to significantly increase (*p *< 0.001) when quality monitoring was stated as the reason for collection [26]. Exploration of similar rationale in the UK could also be informative. Well known artefacts such as the imbalance of disease burden and access to services could be incorporated into the rationale. Standardisation of the point of collection, method of collection, phrasing of questions, available responses and answers to frequently asked questions as suggested by Hasnain-Wynia et al would also be beneficial to both healthcare professionals and patients [23].

Most participants agreed general practice was the most favourable point to collect ethnicity data, where patients are less distressed and with 90% of all patient contact been with primary care there are many more opportunities to capture this information [29]. Additionally, existing patients are already acquainted with reception staff and in familiar surroundings. Collecting at the first hospital visit was also thought to be acceptable as a one-off but repeat recording at subsequent visits was not thought necessary, however repeat visits could be used as a verification point. Initiatives such as NHS electronic Summary Care Record enabling the sharing of up-to-date information will not only prove useful for healthcare professionals and reduce delays in treatment but will also ease the burden of repeatedly giving the same information for patients, ethnicity information could easily be incorporated as part of the patient demographics set [30].

Participants also discussed descriptors of ethnicity they thought to be important in healthcare and also important to distinguish between ethnic groups. Language, religion and country of birth were considered to be instrumental especially for this group of South Asians who are very different culturally in spite of having a similar outward appearance and sharing similar genetic information. One group of participants said they would describe themselves as "British Muslims" completely excluding their country of origin as they felt religious beliefs were the most significant indicator of their culture e.g. religion plays a large part in diet and consumption of alcohol and tobacco.

The findings of the focus groups reported here should be of value to healthcare professionals responsible for collecting routine ethnicity data and may help dispel some of the barriers to data collection. Common obstacles encountered by healthcare professionals are a fear of causing offence to patients, feelings of discomfort when asking the questions and not believing the data to be of relevance. Our research shows South Asians in this sample do not mind sharing this data providing they are given a rationale and the data is used to improve services. Additionally, these findings could be used to identify data items which may be of relevance to particular local populations, additional items such as religion and diet could be added and collected as necessary. The overall aim of this work is to empower ethnicity data collection and prompt reports using this data to meet the requirements stipulated by the Race Relation (Amendment) Act 2000 and to assess whether services are currently meeting the needs of the population. This would need much more work to get right but until we have accurate and complete data on ethnicity we can't estimate rates of disease to see which services and whose needs need assessing. Simply knowing the numbers of BME patients using health services alone is not enough.

Incomplete ethnicity data has meant research to date has had little choice but to utilise methodologies such as 1) use of proxy variables where available such as Country of Birth which have distinct limitations, 2) use of name recognition software such as Nam Pehchan and SANGRA where applicability is limited to South Asians, 3) data linkage has proved useful, 4) sensitivity analyses and 5) multiple imputation or 6) conduct studies tailored to specific populations [31-37]. Landmark reports such as 'Cancer incidence and survival by major ethnic group, England, 2002-2006' produced by the National Cancer Intelligence Network are based upon incomplete data despite linking HES and national cancer registry datasets to form the National Cancer Data Repository [1]. Sensitivity analyses were conducted to assign ethnicity to the 24% of patients with missing ethnicity but crude procedures like this often lead to results that are difficult to interpret. Ryan et al reported inadequacies in both name recognition software and use of census data (ethnic distribution of area of residence) when applied to cancer registry records [38].

Downing et al opted for the more sophisticated multiple imputation in their investigation of the relationship between ethnicity and breast cancer incidence and survival as did the Office for National Statistics in their study of infant mortality for England and Wales by ethnic group [37]. However, multiple imputation is based upon untestable assumptions, in cases where ethnicity data is not missing at random multiple imputation is inappropriate e.g. missing data is concentrated in certain ethnic groups.

Further research is needed into the perceptions and experiences of ethnicity data collection in a broader range of UK ethnic groups e.g. Black Caribbeans, Black Africans, Chinese, Whites and particularly those of the rapidly growing mixed group for whom the question of ethnic group is particularly tricky.

## Conclusion

It is recognised that ethnicity data collection in the UK has historically been of poor quality. Comprehensive and validated ethnicity data collection is essential if we are to reduce inequalities in health and access to healthcare services. In order to improve ethnicity data collection, the provision of training is fundamental in order to increase awareness and promote the importance and utility of recording ethnicity data for all staff that collect/use the data. Ideally, ethnicity should only be collected once by GP or at first hospital visit and linked through healthcare databases and verified at subsequent points of contact. Data collection should be extended to collect additional items such as language, religion and country of origin/birth to account for cultural differences. Only once we have complete and validated ethnicity data can we know the true extent of disparities in healthcare and devise appropriate strategies to combat them. Reducing health inequalities and tailoring current services to meet the needs of BME groups wholly depend upon having accurate and complete ethnicity, without this information we will remain blind to the size and depth of the problem, as a consequence patients with no data will inevitably be left behind.

## Competing interests

The authors declare that they have no competing interests.

## Authors' contributions

GI was the main researcher and contributed to the design and co-ordination of this study in addition to conducting the fieldwork, analysis and drafted the manuscript. MRDJ, AS, SW and AG contributed to the design and co-ordination of this project, interpretation of the data and revision of the manuscript. JAD was the Chief Investigator of the study and contributed to the design and co-ordination of this project, interpretation of the data and revision of the manuscript. All authors have read and approved the manuscript.

## Pre-publication history

The pre-publication history for this paper can be accessed here:

http://www.biomedcentral.com/1471-2458/12/243/prepub

## Supplementary Material

Additional file 1 **Focus group topic guide**. Click here for file
