# Supplementary material for: UK ethnicity data collection for healthcare statistics: the South Asian perspective
Source: BMC Public Health. 2012 Mar 27;12:243. doi: 10.1186/1471-2458-12-243 (PMC3339513; doi:10.1186/1471-2458-12-243)
Supplement: Additional file 1 — Focus group topic guide. [file 1471-2458-12-243-S1.PDF]

Box 1: Focus group topic guide

1. **General opinions on the collection of ethnicity**
   - Do you think accurate recording is important?
   - What do you think it can be used for?
   - Any objections/worries about providing this data?

2. **Experiences of providing ethnicity information**
   - General discussion THEN Focus down on healthcare situations
   - Does anyone know people who have been asked this in relation to 'health research'
   - Does anyone know if the Cancer clinics ask these questions?

3. **Categories used in practice (provide examples on sheets)**
   - Census
   - Hospital admissions
   - GP data
   - Other
   - What categories would you like – how would you prefer to describe yourself

4. **What about language, Religion, Culture:**
   - Do people ask, do you offer this information, do you mind.
   - Are there problems with 'stereotypes' (Explain)

5. **How *<u>should</u>* this information be collected (***if it has to be: Note – the 'Race Relations Act' says that public services should so they can 'combat ethnic inequality')*

   - Would you recommend the routine collection at hospital/GP/other?
   - When would be the best time to collect this data (admission/follow-up after you've been to the hospital once)?
   - How should people ask you – and what should they tell you?
   - Has anyone in the group been asked to take part in 'research' at the hospital or their GP? (i.e. medical research) – Can you tell us about it?

Closing comments

- Does it make a difference in the case of a disease like **cancer** – or is it the same for any health matter?

Is there anything else you want to tell us about?
